# Simulation of electron transport during electron-beam-induced deposition of nanostructures

**DOI:** 10.3762/bjnano.4.89

**Published:** 2013-11-22

**Authors:** Francesc Salvat-Pujol, Harald O Jeschke, Roser Valentí

**Affiliations:** 1 Institut für Theoretische Physik, Goethe-Universität Frankfurt, Max-von-Laue-Straße 1, 60438 Frankfurt am Main, Germany

**Keywords:** electron backscattering, electron transport, (F)EBID, Monte Carlo simulation, PENELOPE

## Abstract

We present a numerical investigation of energy and charge distributions during electron-beam-induced growth of tungsten nanostructures on SiO_2_ substrates by using a Monte Carlo simulation of the electron transport. This study gives a quantitative insight into the deposition of energy and charge in the substrate and in the already existing metallic nanostructures in the presence of the electron beam. We analyze electron trajectories, inelastic mean free paths, and the distribution of backscattered electrons in different compositions and at different depths of the deposit. We find that, while in the early stages of the nanostructure growth a significant fraction of electron trajectories still interacts with the substrate, when the nanostructure becomes thicker the transport takes place almost exclusively in the nanostructure. In particular, a larger deposit density leads to enhanced electron backscattering. This work shows how mesoscopic radiation-transport techniques can contribute to a model that addresses the multi-scale nature of the electron-beam-induced deposition (EBID) process. Furthermore, similar simulations can help to understand the role that is played by backscattered electrons and emitted secondary electrons in the change of structural properties of nanostructured materials during post-growth electron-beam treatments.

## Introduction

Electron-beam-induced deposition (EBID) [1–3] is a suitable method for the template-free fabrication of nanostructures. Molecules of a precursor gas are injected into a high- or ultra-high-vacuum chamber and are dissociated by a focussed electron beam of 1–50 keV into a volatile fragment, which is evacuated by the vacuum system, and a non-volatile fragment, which is progressively adsorbed on a substrate. The latter leads to the growth of a nanostructure at the focus of the beam. In general, the obtained deposits exhibit a granular structure that consists of nanometer-sized metal crystallites, which are embedded in an insulating matrix.

There are three main interactions that determine the growth of nanostructures in the EBID process: (1) the substrate–precursor interaction, (2) the electron–substrate interaction and (3) the electron–precursor interaction. In this work we concentrate on the electron–substrate interaction and our results have some implications for the electron–precursor interaction. Existing theories for the EBID process [4] mainly consist of equations for the deposition rate, which can either be solved analytically under simplifying assumptions or in a more general form by using Monte Carlo simulations. However, there is no theory that addresses the multi-scale nature of the EBID process, including microscopic and mesoscopic length and time scales, from ultra-fast (non-equilibrium processes that last for femtoseconds) to relatively slow (growth and relaxation processes that require nanoseconds or even microseconds).

In this work we focus on the mesoscopic length scale and present a detailed numerical study of the distribution of energy and charge that occurs under EBID conditions. The study is not only relevant for EBID, but it is also a first step to understanding aspects of other experimental techniques including, e.g., the effect of backscattered electrons in the change of structural properties in direct and oxygen-assisted electron-beam post-growth nanostructure treatments [5–6]. We consider various geometric settings as well as different materials relevant for the EBID growth of nanostructures. For our simulations we use the Monte Carlo code for radiation transport PENELOPE [7], in which a statistical set of particle trajectories is sampled in homogeneous materials. In this context, we provide an overview of the aspects of EBID nanostructure growth that can be studied in detail from a mesoscopic point of view by using well-established radiation-transport simulation techniques for amorphous media [8–9]. Recently, practical Monte Carlo simulations of EBID nanostructure growth have been reported [10–13] on the basis of simplified transport models based, e.g., on the Rutherford cross section or on a plural-scattering scheme. The latter averages the inelastic scattering of electrons in solids by using the continuous slowing-down approximation. In this approximation only the energy loss per unit path length is considered and energy fluctuations are not captured. In the present work we sample inelastic interactions in detail, i.e., on a per-interaction basis without employing a condensed simulation scheme, and we restrict our considerations to the interaction of the primary electrons with the substrate and the nanostructure at different stages of its growth.

The precursor gas we consider throughout this study is tungsten hexacarbonyl, W(CO)_6_, and the corresponding deposits W*_x_*C*_y_*O*_z_*, i.e., amorphous tungsten oxycarbides with varying carbon and oxygen contents. W(CO)_6_ belongs to the class of organometallic compounds that are well established for the EBID process [14–16]. It has been studied in detail by mass spectrometry [17–19] and photoelectron or photoionization spectroscopy [20–22], which yield appearance energies of ionic fragments as well as approximate internal energy distributions after electron ionization. The main advantage of using this precursor gas is that the tungsten metal content in the deposits can be widely varied so as to cover a wide range of electronic properties, from insulating to metallic [16,23]. Our aim is to determine a spatially resolved picture of the growth conditions created by the electron beam within and above a SiO_2_ substrate as well as within and above W*_x_*C*_y_*O*_z_* deposits of various thicknesses.

## Description of the simulation

**Figure 1:**
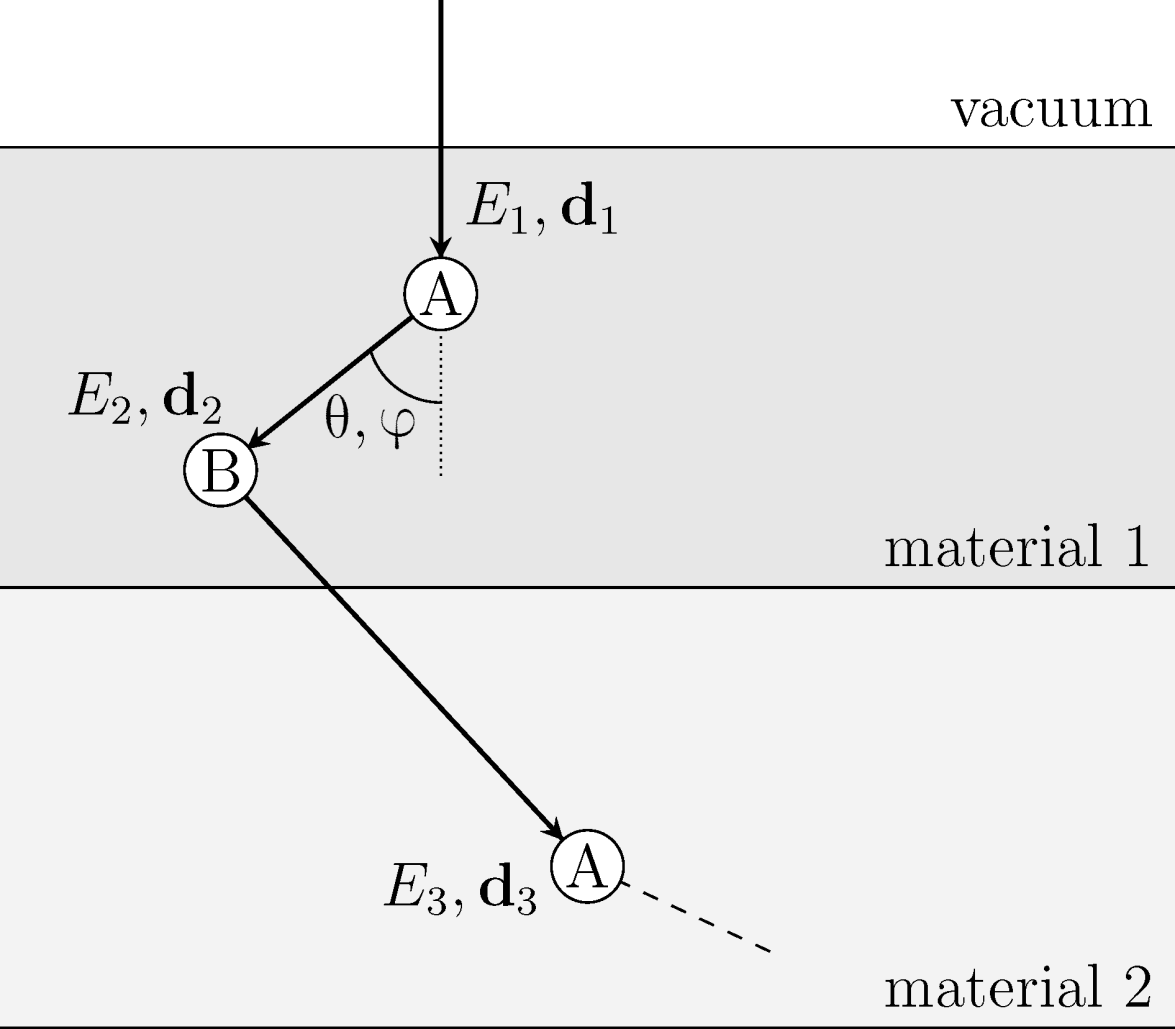
Schematic representation of a random trajectory generated by PENELOPE [7]. The trajectory is determined by the path lengths *s* that determine the position **r***_n_* of the next scattering event, by the types of event, and by the energies *E_n_* and the directions **d***_n_* after the event.

The Monte Carlo method for the simulation of radiation transport is a numerical means of solving the Boltzmann transport equation in an arbitrary geometry. The computer code system PENELOPE yields trajectories of primary and secondary particles according to state-of-the-art interaction cross sections on sample geometries constructed by positioning a set of well-defined homogeneous bodies in space. Random trajectories are generated as follows [7]: particles are characterized by their position vector **r** = (*x*,*y*,*z*), energy *E* and a direction-of-flight unit vector **d** = (*u*,*v*,*w*), where *u*, *v*, and *w* are the direction cosines. A particle trajectory is represented as a series of states (**r***_n_*,*e_n_*,**d***_n_*), where *n* labels the scattering event at **r***_n_* that leads to an energy *E_n_* and a direction **d***_n_* (see below in Figure 1). Several random variables are sampled from their respective probability distribution functions. The length of the free path to the next collision, *s*, is sampled from an exponential distribution with a total mean free path λ*_T_* by using a random number ξ uniformly distributed in the interval (0,1),

[1]
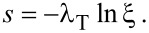


The interaction type at the new position is sampled as follows: Let us consider interactions of type A and B with the respective total cross sections σ_A_ and σ_B_. The interactions of type A and B are sampled with the probabilities

[2]
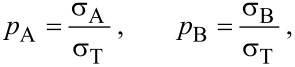


respectively, where σ_T_ = σ_A_ + σ_B_ is the total interaction cross section. The polar scattering angle θ and the energy loss *W* are sampled from a distribution with azimuthal symmetry,

[3]
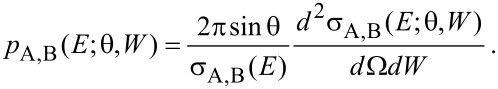


Finally, the azimuthal scattering angle is sampled from a uniform random number ξ as φ = 2πξ.

The PENELOPE code [7] uses a relatively sophisticated interaction model that is devised for energies above a few hundred eV. Differential cross sections for elastic scattering were calculated with the state-of-the-art relativistic partial-wave calculation code ELSEPA [24]. Inelastic interactions are described by means of the plane-wave Born approximation, which uses a schematized generalized-oscillator-strength model that is fitted to reproduce the stopping power obtained from the asymptotic Bethe formula at high energies.

**Figure 2:**
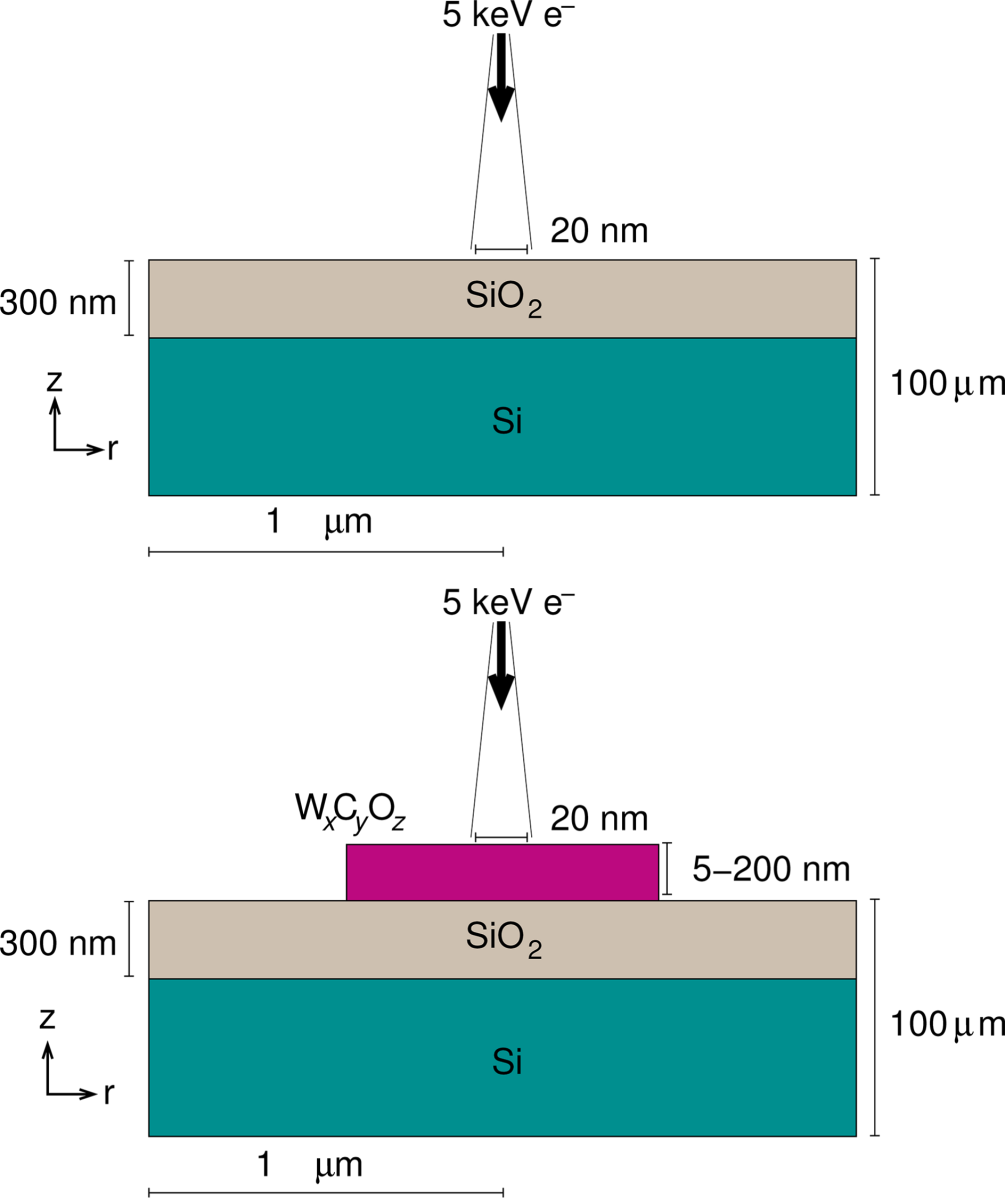
Cylindrical sample geometries used in the simulations. Top: A 300 nm thick amorphous SiO_2_ substrate is placed on top of a Si wafer and is irradiated with 5 keV electrons. Bottom: W*_x_*C*_y_*O*_z_* deposits of thicknesses between 5 and 200 nm and of densities and compositions as given in Table 1 is placed on top a 300 nm amorphous SiO_2_ layer, which in turn is placed on top of a Si wafer. A conical electron beam with a spot size of 20 nm on the sample is used, the point source being located 1 cm in vacuum above the center of the sample. The corresponding beam aperture is 5.73 × 10^−5^ degrees. A radius of 100 nm is chosen for the deposit.

In our study it is convenient to reduce the problem to two spatial dimensions by assuming a geometry with cylindrical symmetry. We perform studies for two classes of sample geometries: (a) a 300 nm thick layer of amorphous SiO_2_ with a density of 2.32 g/cm^3^ is placed on top of a Si wafer with a density of 2.33 g/cm^3^ in order to study the initial conditions of the EBID growth process. We refer to this sample geometry briefly as the “substrate”. (b) Structures corresponding to intermediate EBID deposits are constructed in order to study the conditions for further growth in the EBID process, in which deposited layers of different thicknesses (from 5 nm to 200 nm) are placed on top of the substrate surface. Density and composition of these model structures are set in accordance with six different experimentally realized EBID structures [16]. While the composition in terms of atomic percent is taken from [16], the densities were determined in [23] by predicting approximate crystal structures at these given compositions by using evolutionary-algorithm-based crystal structure prediction. The composition and densities of the deposits are listed in Table 1. In both cases, an electron beam of 5 keV with a spot size of 20 nm diameter impinges perpendicularly on the surface. In practice, the electron beam is rastered on the substrate, so that the extension of the deposited nanostructure can be larger than the electron-beam spot size. Thus, a radius of 100 nm has been used for the deposited nanostructure. The linear range of 5-keV electrons in Si and W is about 0.4 μm and 0.1 μm, respectively. Thus, in order to ensure that virtually no electrons leave the simulation geometry through the lateral bounds (the direction perpendicular to the incoming direction), a cylinder radius of 1 µm has been set. See Figure 2 for an illustration.

**Table 1:** Composition of the six amorphous tungsten oxycarbide deposits considered in this study, following [16] and given in terms of atomic percent (atom %). They are sorted by increasing density, which was determined in [23] (see text).

composition approximant	density (g/cm^3^)	W (atom %)	C (atom %)	O (atom %)

WC_2.5_O	7.9	22.6	56.0	21.4
WC_3.33_O_0.67_	8.7	19.0	67.1	13.8
WC_1.4_O_0.8_	9.1	31.8	44.4	23.8
WCO_0.71_	10.0	36.9	35.6	27.5
WC_1.33_O_0.67_	10.4	34.0	44.3	21.7
WC_1.75_O_0.75_	10.6	27.5	50.4	22.1

The material cross sections in our calculations are approximated as an averaged weighted sum of the atomic cross sections that correspond to a given composition (incoherent sum of scattered intensities). Thus, they neglect chemical binding effects. Energetic electrons can scatter either elastically, when the quantum state of the scatterer remains unaltered and the direction of the projectile changes, or inelastically, when electronic excitations or ionizations take place through the different energy and momentum transfer channels available. As the electrons evolve through the medium, they lose energy in the course of several inelastic interactions. The lost energy is either absorbed by the medium through local excitations, which are allowed to relax through the emission of photons, or through ionization of the sample, which leads to the build-up of a localized positive charge in the material and to new particles, thus leading to a “shower” of particles. If an electron crosses a boundary into an adjacent material, its trajectory history is stopped at the other side of the interface and restarted with the new material transport properties. This can be done any time, since electron trajectories are modelled as Markov processes (the future of the trajectory is dependent only on the present state, and not on the past). The trajectory history of an electron is stopped when its energy drops below 50 eV. The electron is then considered to be absorbed by the medium, and to be contributing to the build-up of a localized negative charge in the material. We choose an absorption energy of 50 eV because we are neglecting binding effects in the material, and furthermore, elastic and inelastic cross sections derived from atomistic models carry large uncertainties already for energies below a few hundred eV. The same absorption energy is used for the secondary electrons that are generated in the shower. Finally, to obtain our simulation results, we have sampled 10^8^ trajectories.

## Results

**Figure 3:**
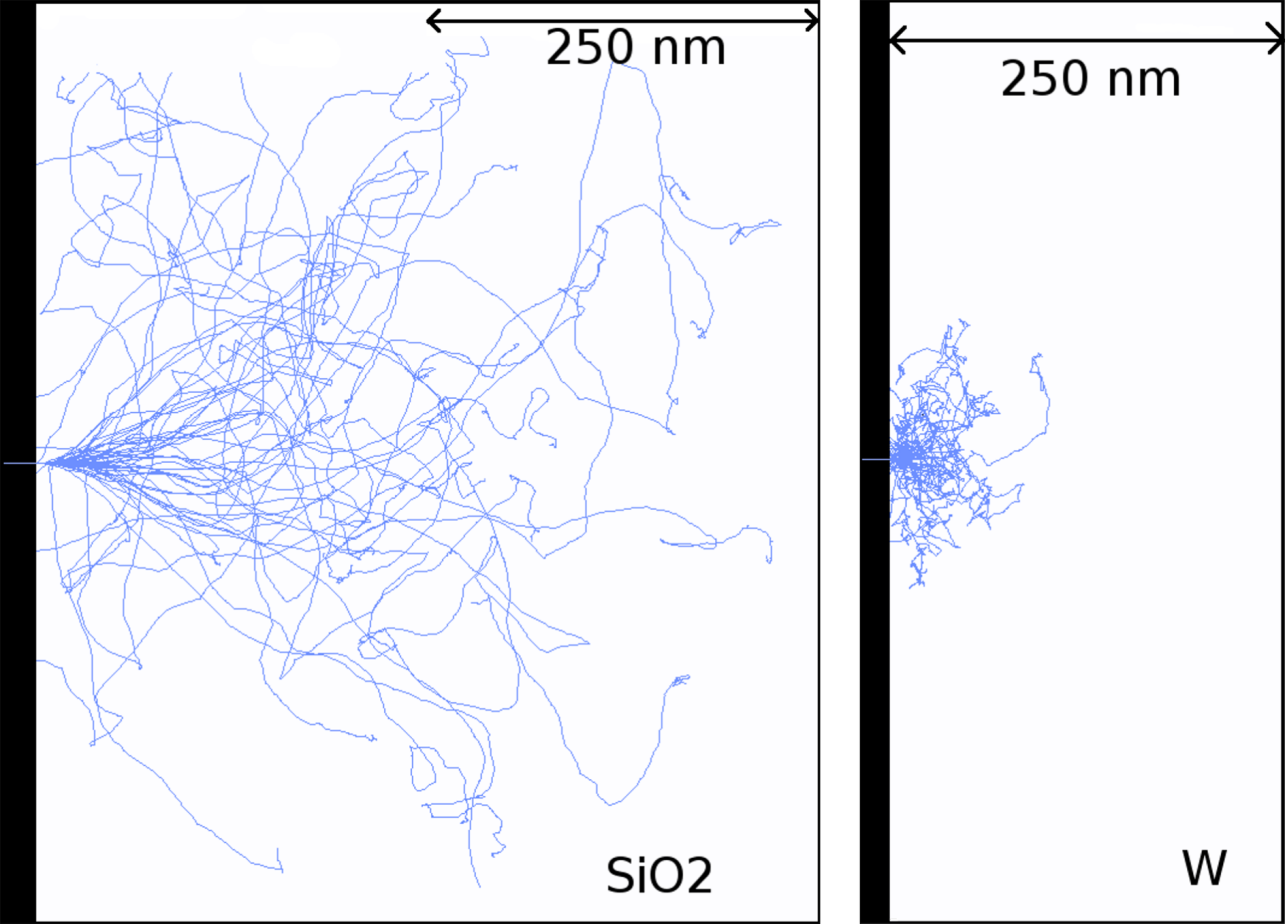
Snapshot of 50 simulated electron trajectories in the SiO_2_ substrate (left) and in the nanostructured deposit material W (right, representative for material deposit). The width of the screenshot windows corresponds to 500 nm.

**Figure 4:**
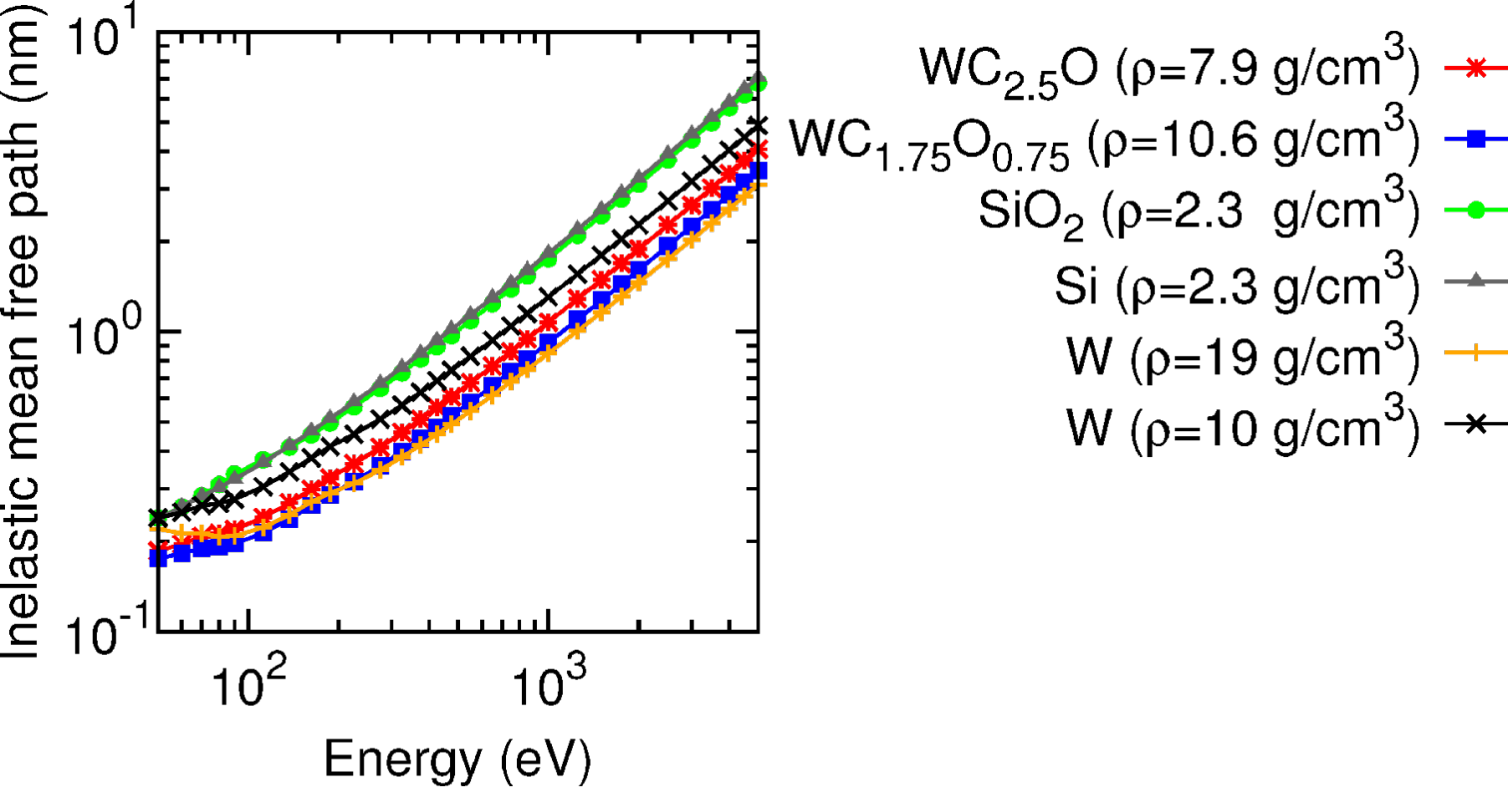
Inelastic mean free paths for the relevant materials in this work presented in the usual log–log scale. The tungsten oxycarbide compositions WC_2.5_O and WC_1.75_O_0.75_ correspond to the samples with the lowest and highest density in Table 1, respectively. The two curves with cross symbols show the variation due to a density change only.

To provide a first visual insight into the electron transport process in the substrate and in the deposited nanostructures, Figure 3 displays a simulated shower of 5-keV-electron trajectories impinging perpendicularly on a 500 nm thick slab of SiO_2_ (left-hand side, substrate material) and on a 500 nm thick slab of pure W (right-hand side, deposit material), respectively. We consider pure tungsten as a representative material of the different deposits for practical reasons. This choice is reasonable inasmuch as the average distance between consecutive inelastic collisions [inelastic mean free path (IMFP)] of electrons in W and in the considered nanostructure materials are very similar in the energy window of interest (see Figure 4). Note that in the SiO_2_ substrate the beam is completely attenuated at a depth of approx. 500 nm, whereas in W this depth is reduced to approx. 150 nm. Indeed, the IMFP of electrons in SiO_2_ is roughly by a factor 2–4 larger than the IMFP of electrons in W (or any of the six considered deposit materials), as shown on the left-hand panel of Figure 4. Thus, we conclude that in the early stages of the nanostructure growth, i.e., when the thicknesses are much smaller than ca. 150 nm, the electron beam hits both the thin deposit and the substrate. The energy and charge deposition processes are therefore dictated by the transport characteristics of both the deposit and the substrate. On the other hand, for nanostructure thicknesses exceeding approx. 150 nm, the deposition of energy and charge takes place almost exclusively in the nanostructure without affecting the substrate. A similar analysis has been carried out in [13]. Experimentally, similar conclusions were drawn from current measurements [25].

**Figure 5:**
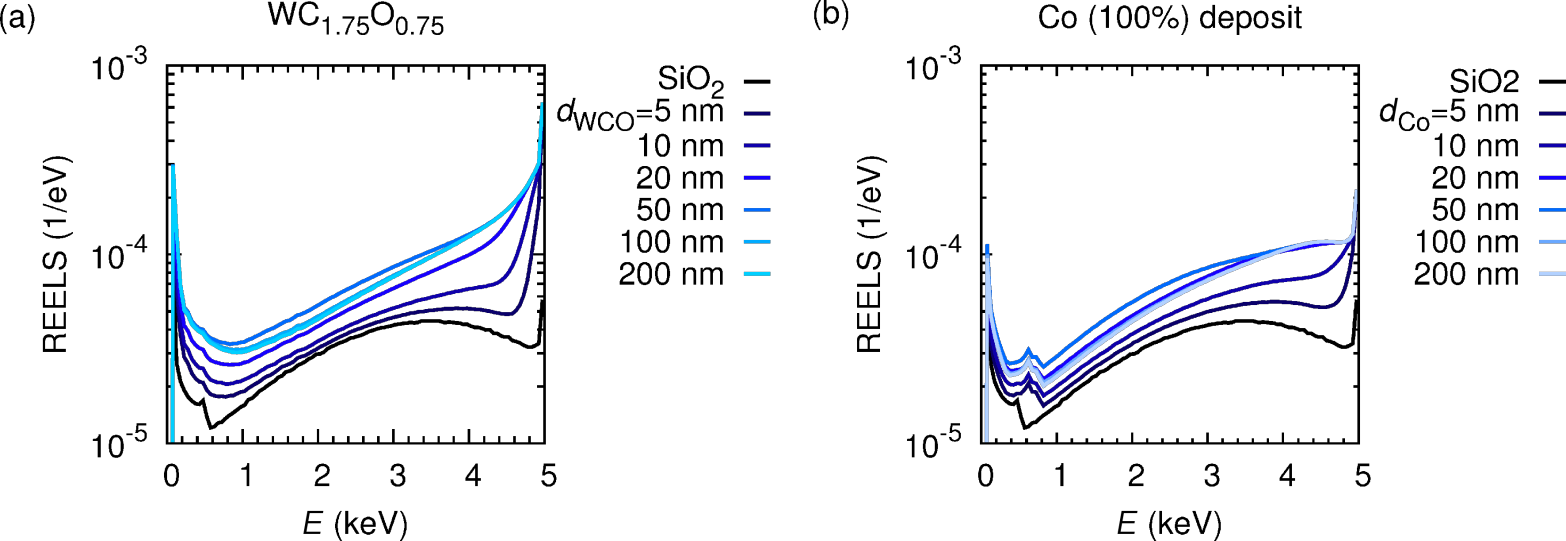
(a) Distribution of electrons backscattered and emitted into the vacuum from the substrate in absence of a deposit (black curve) and from the substrate with a deposit of thickness *d*_WCO_ in the range from 5 to 200 nm (blue curves), which consists of the material corresponding to the composition WC_1.75_O_0.75_ (see Table 1). (b) Same as panel (a) for a deposit of pure Co. Notice that the ordinates are in a logarithmic scale, whereas the abscissas are in a linear scale. The acronym REELS stands for reflection electron-energy-loss spectrum.

Figure 5a displays the energy distribution of electrons that backscattered and emitted per incoming electron from the substrate, (darkest curve) and from deposits of increasing thicknesses *d*_WCO_ on top of the substrate [dark blue curve, *d*_WCO_ = 5 nm, through light blue curve, *d*_WCO_ = 200 nm]. Notice that for thin deposits the spectral features of the substrate are merely smeared out, owing to the fact that only few inelastic interactions take place in the thin deposit. For increasing deposit thicknesses, the transport in the substrate plays an increasingly marginal role. Thus, for thick deposits the spectral features of the substrate vanish and the spectral features of the deposit prevail. This explains the saturation behavior of the curves that correspond to *d*_WCO_ = 100 nm and *d*_WCO_ = 200 nm, in which electrons are very unlikely to even reach the substrate, in accordance with the discussion of Figure 3. It is interesting to note that the intensity in the energy distribution of backscattered electrons increases with the sample thickness. Indeed, on the one hand the elastic backscattering coefficient increases with the atomic number. This leads to the observed increase in the elastic peak at 5 keV, because the substrate consists of Si and O (atomic numbers *Z* = 14 and *Z* = 8, respectively) whereas the deposit material contains W (*Z* = 74). On the other hand, the IMFP is inversely proportional to the material density, so that a denser deposit on a comparatively light substrate implies an increase in the number of energy losses per unit path length compared to those that would take place in the substrate alone. This justifies the factor of about 2 between the curves corresponding to the (thick) deposit and the substrate. Thus, under the assumption that the presence of a large number of electrons (slow or fast) enhances the dissociation rate of the precursor gas molecules adsorbed on the substrate, one can infer the following positive-feedback process: As the deposit thickness grows, so does the number of backscattered and emitted electrons, leading to an improvement in the dissociation rate and, therefore, in the deposition rate of the nanostructure, which in turn leads to a reinforced growth process. A more quantitative description of the change in deposition rate would imply analyzing the separate contributions from backscattered electrons, forward-scattered electrons, and secondary electrons [26].

Two aspects of Figure 5a should be emphasized. (1) In order to further elucidate the dependence of the electron backscattering probability on the atomic number of the deposit material, the simulation was repeated while replacing the deposit with Co, a comparatively lighter material (*Z* = 27). Figure 5b displays the energy distribution of backscattered electrons for different Co-nanodeposit thicknesses, *d*_Co_. Notice that the increase in the elastic-peak intensity is roughly a factor 2 or 3 smaller than for the nanostructure material, which is much heavier. (2) Notice that as the deposit becomes thicker, the intensity of the curves increases monotonically, reaching its maximum for a thickness of about 50 nm and then decreasing slightly into its saturated value for a thickness of 200 nm. The fact that multiple elastic and inelastic interactions take place along the trajectory makes it hard to give a detailed explanation of this effect. Nevertheless, it can be argued that for thicknesses exceeding 50 nm, the fraction of trajectories which reach the substrate becomes negligible and, for thick enough deposits, this fraction approaches zero. Owing to the fact that the mean free paths in the deposit are much shorter than in the substrate, more energy losses take place per unit path length in the deposit than in the substrate. This implies that the thicker the deposit becomes, the larger is the number of electrons which leave the sample after losing most of its energy. This explains, at least qualitatively, the increase and eventual saturation in the low-energy regime of the spectrum (contribution of electrons which leave the sample after losing most of its energy and of emitted secondary electrons), as well as the decrease in intensity in the energy range between 1 and 4 keV (for thick substrate electrons in this regime lose more energy and therefore the spectral intensity shifts to lower energies). Close to the elastic peak, variations with the thickness of the substrate between 50 nm and 200 nm are not visible, since the elastic backscattering probability for the deposit is much larger than for the substrate.

**Figure 6:**
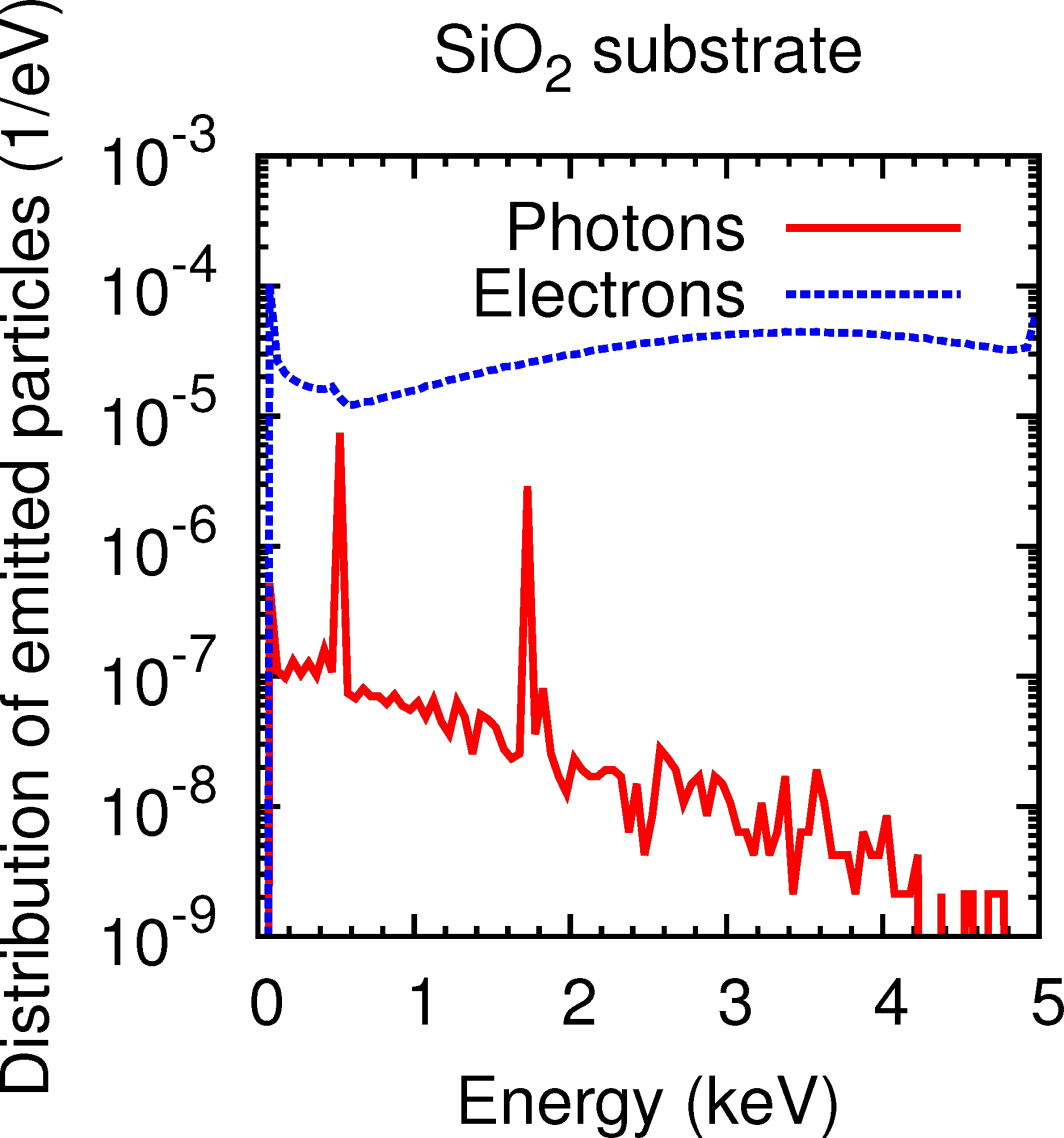
Distribution of electrons backscattered and emitted into the vacuum from the substrate in the absence of a deposit (dashed curve) and distribution of photons emitted into the vacuum (solid curve). Note, that the ordinates are in a logarithmic scale, whereas the abscissas are in a linear scale.

The primary energy of the electrons (5 keV) is high enough to produce inner-shell ionizations in Si and O. Let S0 denote the ionized shell. A second electron from an outer shell, S1, fills the vacancy and, subsequently, two processes are possible: (1) a radiative transition in which a photon is emitted with a characteristic energy *U*_S0_ − *U*_S1_, where *U* denotes the ionization energy of the corresponding shell, or (2), typically more likely, a non-radiative transition in which an electron from an outer shell S2 (which can either coincide with or be less bound than S1) is emitted as an Auger electron with the energy *U*_S0_ − *U*_S1_ − *U*_S2_. The emitted photons might either leave the sample or be absorbed by a target atom, which leads to the emission of photoelectron. Figure 6 displays the distribution of electrons (solid red curve) and photons (dashed blue curve) emitted from the substrate per incoming electron in the absence of a deposit. The peaks in the photon spectrum, superimposed on a Bremsstrahlung background, correspond to the K lines of Si and O, situated at 1739–1835 eV and 523 eV, respectively. Notice that the number of photons emitted per incoming electron is at least two orders of magnitude smaller than the number of emitted electrons. Furthermore, the interaction mean free paths for photons are typically much longer than those for electrons. Thus, the contribution of the emitted photons to the energy and charge deposition processes is presumably negligible, except for the minor photoelectron peak in the electron spectrum of the substrate at 500 eV, superimposed to a contribution from Auger electron emission from O with energies from 478.8 eV to 508.9 eV. A contribution of Auger- or photoelectrons is not observed at 1739–1835 eV, because (1) Auger-electron energies are spread over a few hundred eV and thus do not lead to a well resolved peak and (2) the photoelectric cross section at these energies is one order of magnitude smaller than at 500 eV (cross section data taken from the numerical database of PENELOPE [7]). The photon spectrum was also examined with a deposit on top of the substrate. But no significant deviations regarding the minor role played by photon transport, as demonstrated for the pure substrate, was found.

**Figure 7:**
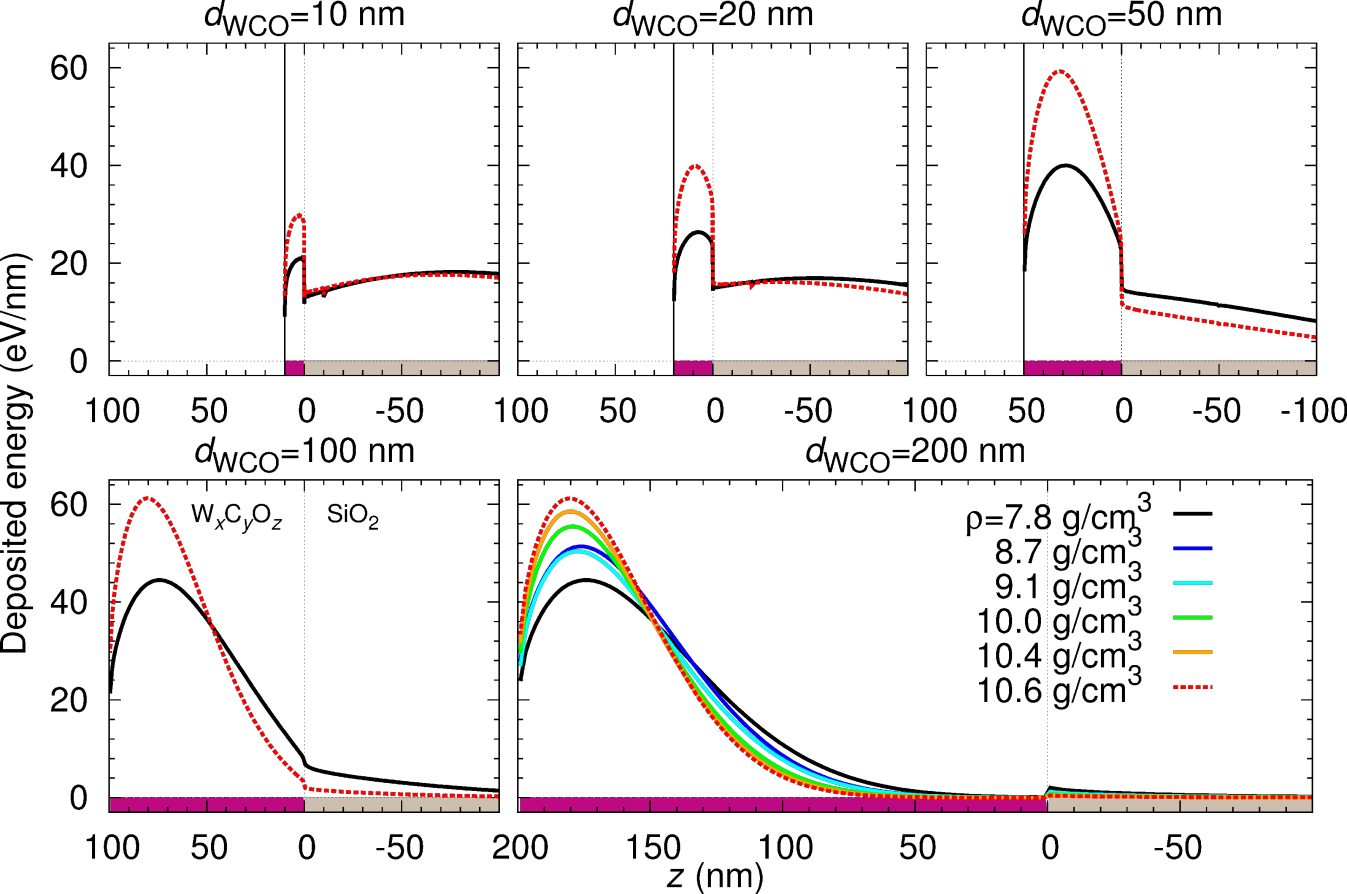
Energy deposited in the system as a function of the depth *z* for the indicated sample thicknesses *d*_WCO_ and for the six nanostructure materials specified in Table 1. The position *z* = 0 corresponds to the deposit-substrate interface; the position *z* = *d*_WCO_ (indicated by a solid vertical line in the three upper panels) corresponds to the deposit-vacuum interface. Notice that, in addition to the density, the composition of the samples varies (see Table 1).

Figure 7 displays the distribution of the energy deposited in the system as a function of depth for sample thicknesses *d*_WCO_ in the range from 10 to 200 nm. Negative depths correspond to the SiO_2_ substrate, whereas positive depths denote the deposit, indicated respectively by the magenta and grey bars (reflecting the color code in Figure 2). The black solid and the dashed red curve correspond to WC_2.5_O (lowest density sample) and WC_1.75_O_0.75_ (highest density sample), respectively. The panel corresponding to *d*_WCO_ = 200 nm additionally shows the deposited energy for samples with intermediate values of density. It is clear that the deposited energy per unit depth is much higher in the deposit than in the substrate, since the IMFP is by a factor of aprox. 2 shorter in the deposit than in the substrate, and thus energy-loss events take place more often in the deposit than in the substrate. This also explains the discontinuous jump at the deposit–substrate interface.

**Figure 8:**
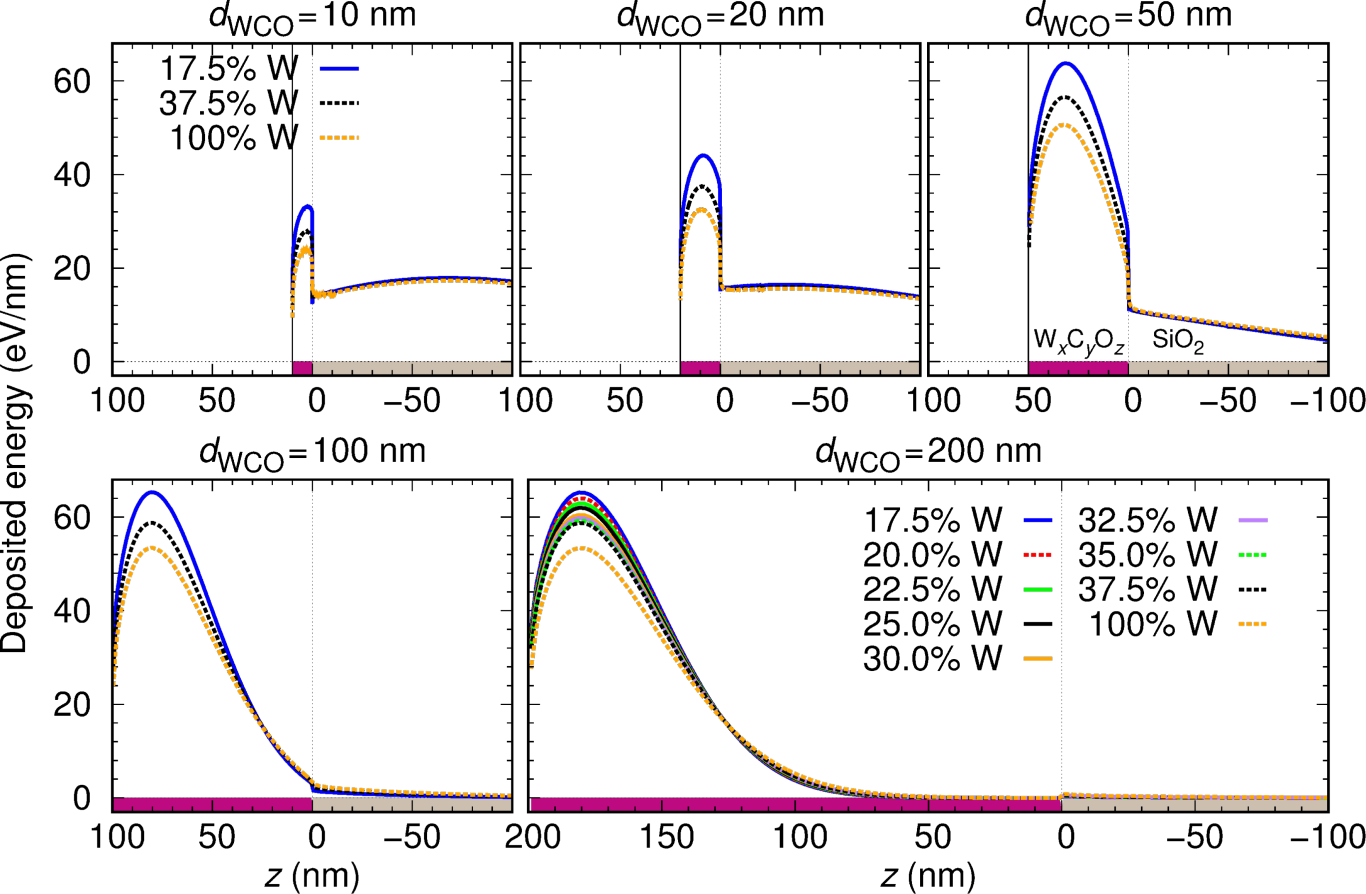
Same as Figure 7 for a fixed deposit density (*ρ* = 10.6092 g/cm^3^) and a variable tungsten content.

**Figure 9:**
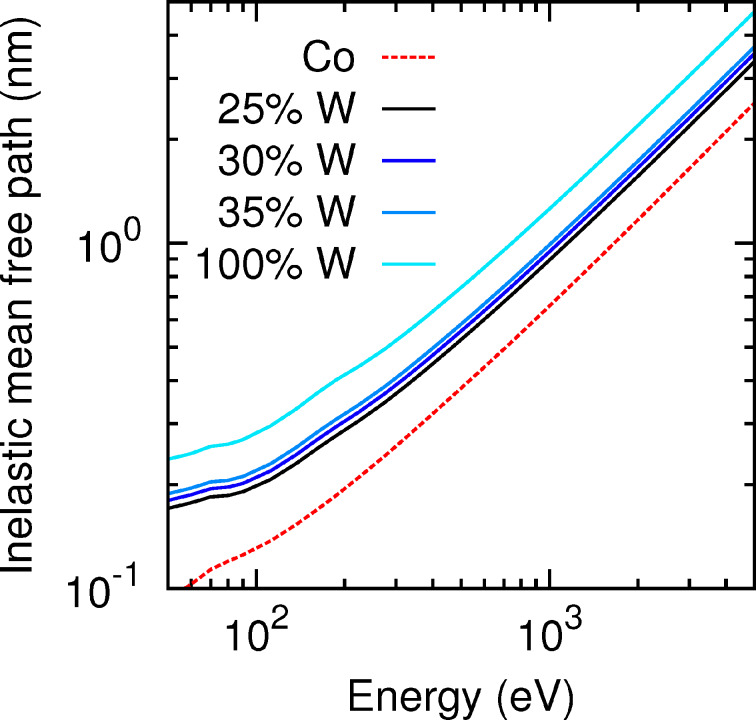
IMFP in the different deposits with fixed density (*ρ* = 10.6092 g/cm^3^) and a variable tungsten content.

**Figure 10:**
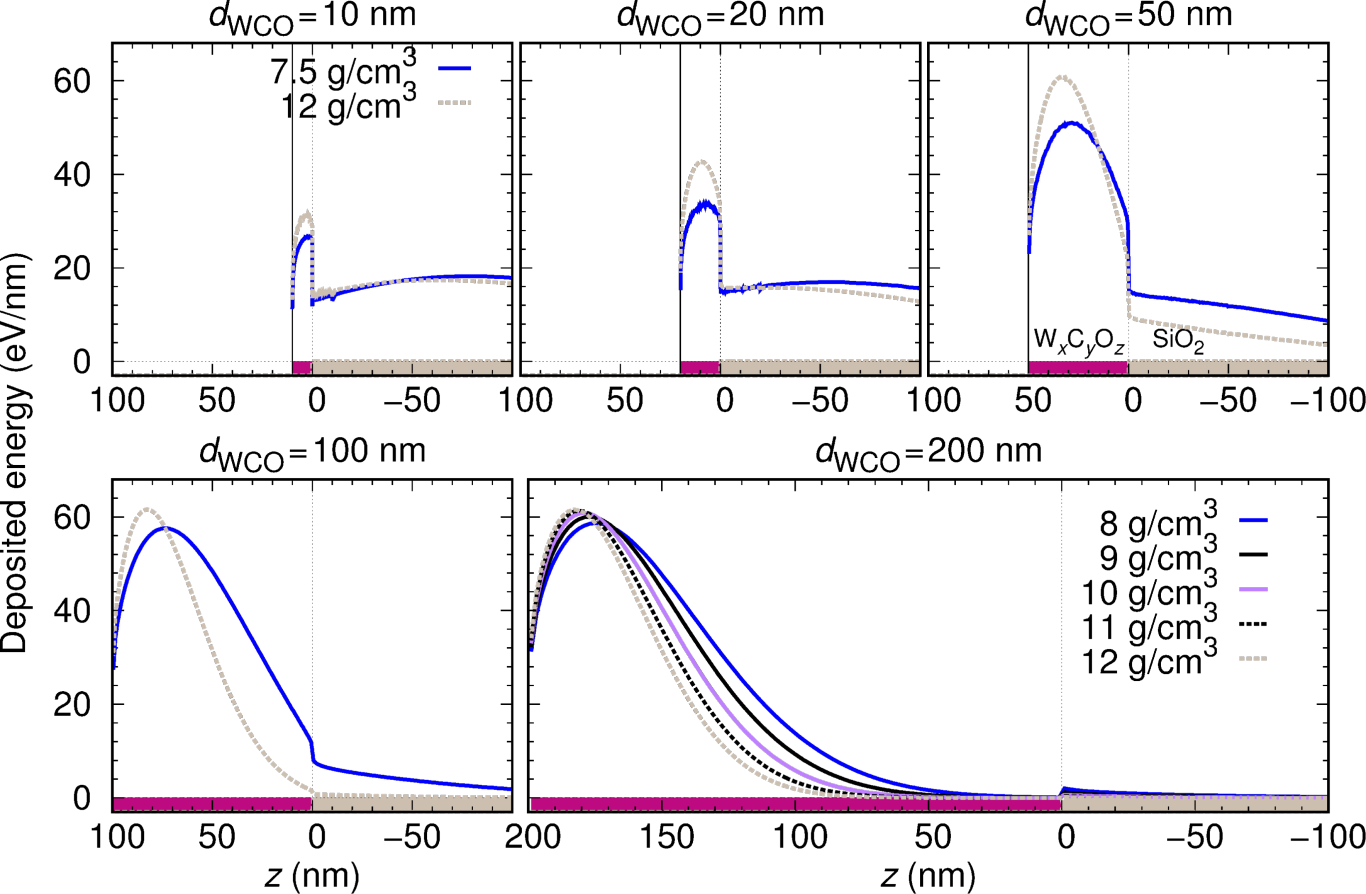
Same as Figure 7 for a fixed composition (27.5% W, 50.4% C, 22.1% O) and a variable density.

**Figure 11:**
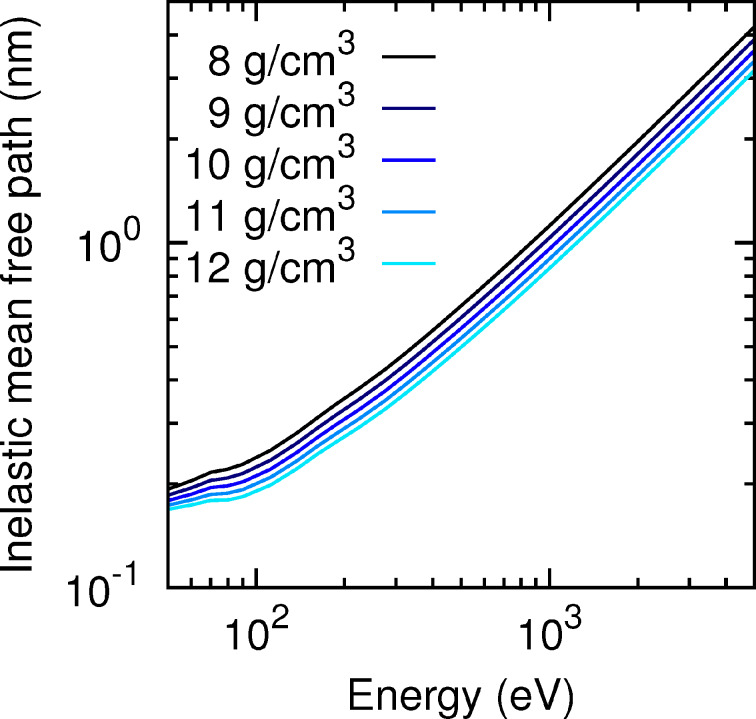
IMFP in different deposits with fixed composition (27.5% W, 50.4% C, 22.1% O) and a variable density.

It should be noted that, whereas the density increases linearly from WC_2.5_O to WC_1.75_O_0.75_, the tungsten content does not exhibit a clear trend (see Table 1). In order to separately demonstrate the effect of density and W-content variations on the distribution of deposited energy, we have considered the following artificial material variations. On the one hand, we have taken a sample with a fixed density *ρ* = 10.6092 g/cm^3^ (corresponding to WC_1.75_O_0.75_) and have varied its W content from 17.5% to 37.5% in steps of 2.5% (covering the range of W contents in Table 1), while decreasing both the C and the O contents by 1.25% at each step. We have also considered the extreme case of 100% W content. The distribution of the deposited energy as a function of the depth is shown in Figure 8, and the corresponding IMFPs are displayed in Figure 9. On the other hand, we have taken a sample with a fixed W content (27.5% W, 50.4% C, 22.1% O, which corresponds to WC_1.75_O_0.75_) and have varied its density from 8 g/cm^3^ to 12 g/cm^3^ (covering the range of densities given in Table 1). The distribution of the deposited energy and the corresponding IMFPs are shown in Figure 10 and Figure 11, respectively. Comparing Figure 8 and Figure 10 we conclude that variations in the density influence the energy deposition process in the nanostructure much more strongly than variations in the W content (in the considered variation intervals of these parameters). This can be best observed in the case of the sample with thickness *d*_WCO_ = 200 nm for *z* = 50–150 nm.

**Figure 12:**
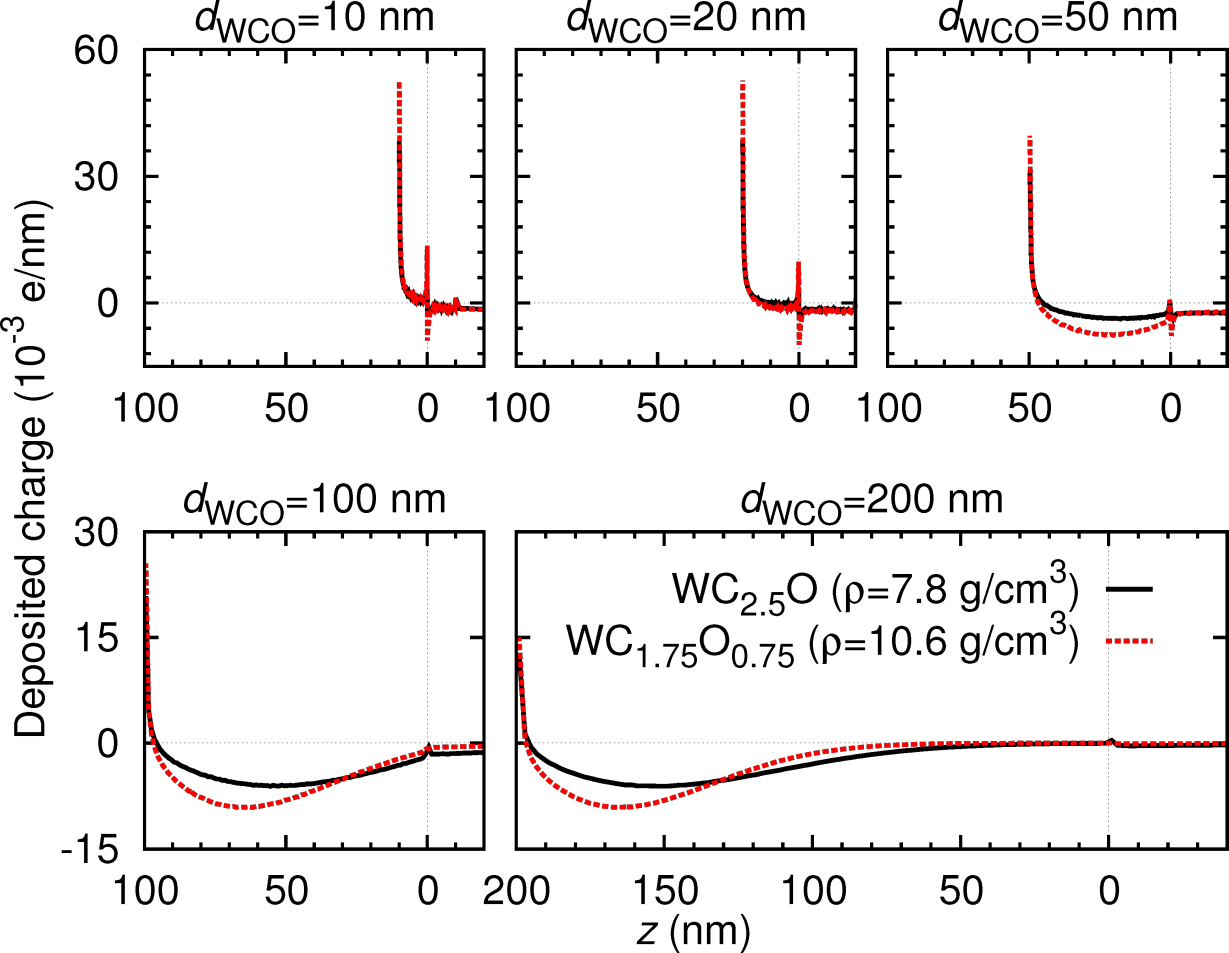
Charge deposited into the system as a function of the depth *z* for the indicated sample thicknesses *d*_WCO_ and for the six nanostructure materials specified in Table 1 (compare with Figure 7).

In practice, sample charging effects in the EBID process cause only a minor repulsion of the electron beam (observed as a slight drift in the monitoring images), which can be easily corrected by applying appropriate beam-deflection voltages. Nevertheless it is interesting per se to examine the spatial distribution of the charge deposition process induced by the incoming beam, if only to better delimit the spatial region that is probed and affected by the beam. Figure 12 displays the distribution of charge deposited per unit path length for a deposit thickness *d*_WCO_ ranging from 10 to 200 nm in WC_2.5_O and WC_1.75_O_0.75_ (solid black and dashed red line, respectively). Calculations were also carried out for samples of intermediate densities but are not shown in the Figure, which displays only the two extreme cases for clarity. Note, that the charge deposited in the nanostructure close to the vacuum interface is positive. This implies that there are more secondary electrons emitted from this region than slow electrons absorbed in it. Indeed, those secondary electrons emitted from the nanostructure into the vacuum do not return, implying that close to the vacuum interface it is more likely to see a lack of electrons than an absorption of slow electrons. Deeper into the nanostructure, the absorption of slow electrons becomes more likely: Secondary electrons are more likely to become absorbed than to reach the interface into vacuum. This leads to the observed decrease in the deposited charge, which becomes even negative when the nanostructure is thick enough so that all generated secondary electrons are eventually absorbed in it. Regarding the deposit–substrate interface, two aspects should be considered. On the one hand, the primary electron loses less energy in the SiO_2_ substrate than in the nanostructure material, so that less secondary electrons are generated per unit path length in the substrate. On the other hand, secondary electrons from the nanostructure cross the interface into the substrate. The number of slow electrons moving from the nanostructure into the substrate is larger than the number of slow electrons moving in the opposite direction. This leads to the observed increase in positive (negative) charge in the nanostructure (substrate) side of the interface.

**Figure 13:**
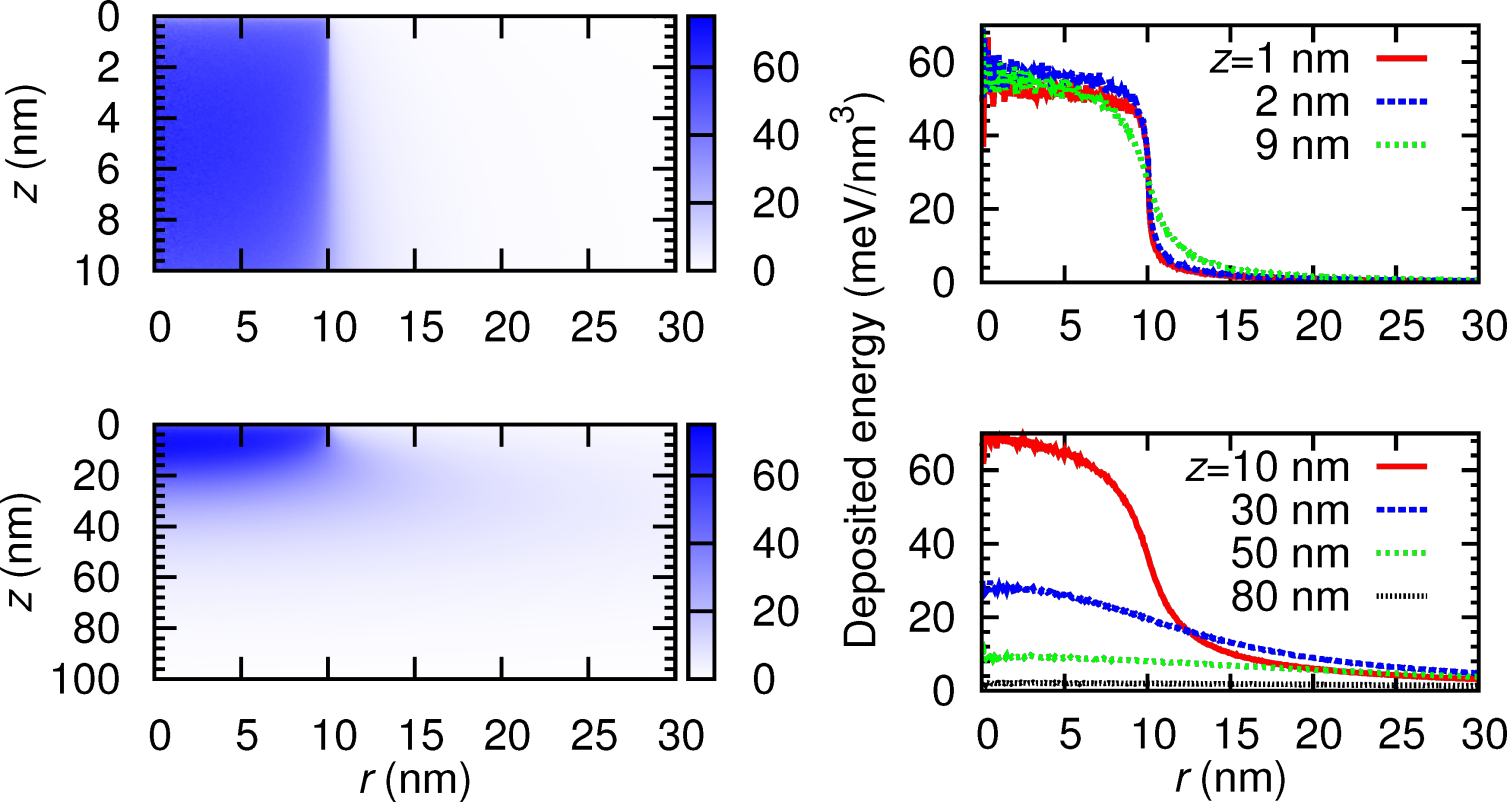
Distribution of energy deposited in WC_1.75_O_0.75_ as a function of the depth *z* and the radial coordinate *r*. The two upper (lower) panels correspond to a sample thickness *d*_WCO_ = 10 nm (*d*_WCO_ = 100 nm). The right-hand-side panels display cross sections of the distribution at the indicated depths *z* below the deposit-vacuum interface.

**Figure 14:**
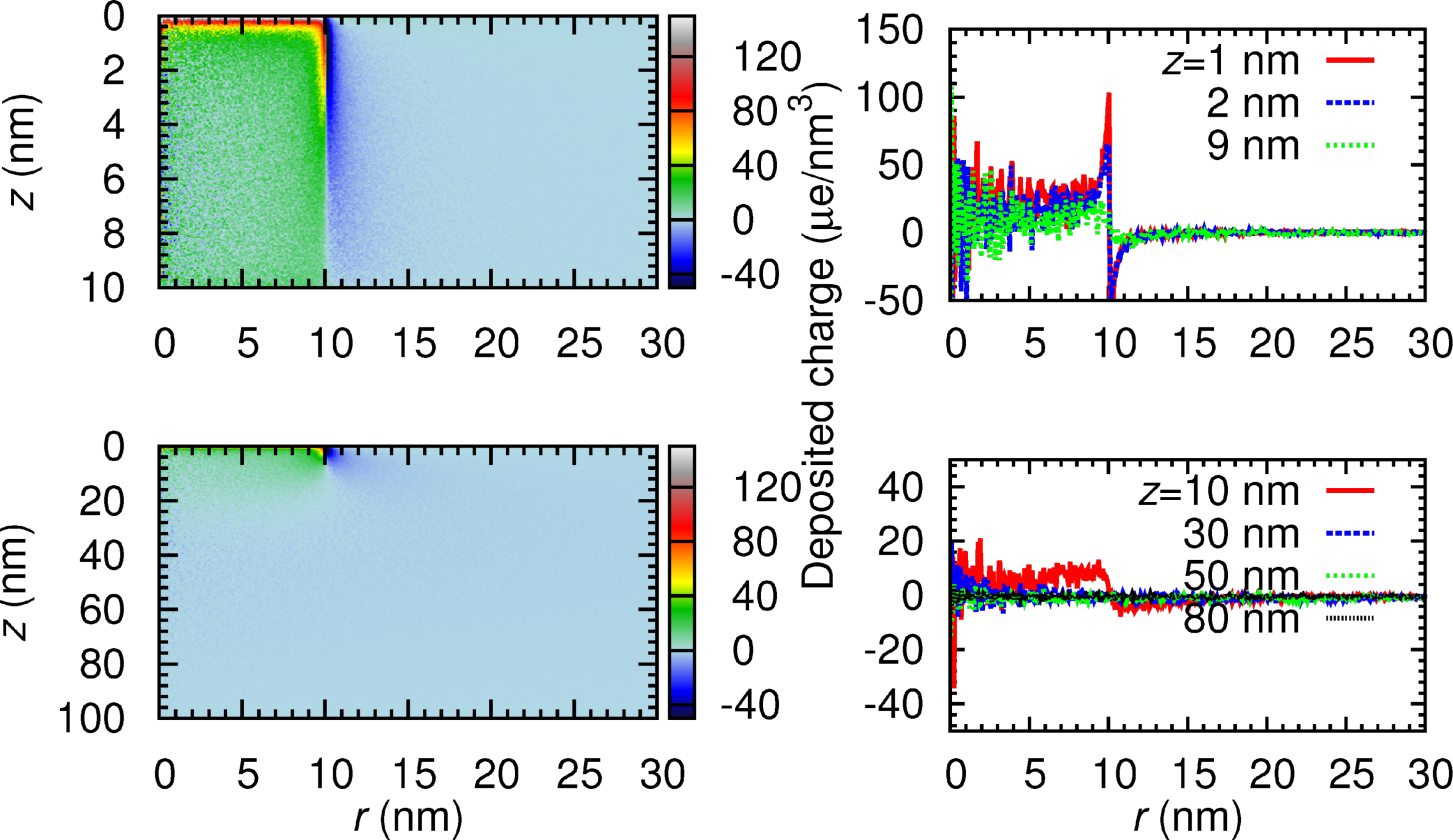
Same as Figure 13 for the charge deposited in WC_1.75_O_0.75_.

Finally, Figure 13 and Figure 14 display, respectively, the distribution of deposited energy and charge as a function of the depth and the radial coordinate in WC_1.75_O_0.75_ (highest density sample) for nanostructure thicknesses of 10 and 100 nm. The panels on the right-hand side show cross sections of the distributions at the indicated depths *z*. In these figures one can clearly identify the beam radius of 10 nm. Notice that at radii *r* < 10 nm the deposited charge is positive. In this region, secondary electrons are emitted as a result of the energy loss of the primary electrons. For distances *r* > 10 nm, the deposited charge is negative, meaning that electrons with *E* ≤ 50 eV are absorbed there. These slow electrons are those secondary electrons generated in *r* < 10 nm that move to *r* > 10 nm and are not able to travel further, because they are absorbed.

The distribution of the deposited energy as a function of the depth and of the radial coordinate has an additional value. On one hand, it can be used to derive a temperature distribution for more detailed microscopic simulations (e.g., molecular dynamics) of the EBID process [27]. On the other hand, the deposited energy also contributes to an enhancement of the dissociation of the precursor gas molecules adsorbed on the surface. It is therefore worthwhile to have an accurate estimate for this quantity. The consequences for the growth of the nanostructures are an interesting topic for further research.

## Conclusion

In this work we presented the results of Monte Carlo simulations of the electron transport that provide valuable insight into the charge and energy deposition processes induced by the primary electron beam in the EBID process of W(CO)_6_ nanostructures on SiO_2_ substrates. The simulations highlight the differences in the transport of electrons in the nanostructure and in the substrate. The mean free path between consecutive inelastic interactions in the deposit is a about a factor of 2 smaller than in the substrate, which leads to a beam attenuation after a depth of ca. 500 nm in the substrate material, whereas, in the nanostructure material, the beam is attenuated at much shallower depths of approx. 150 nm. In the early stages of the nanostructure growth (thickness well below 150 nm), a significant fraction of incoming electron trajectories still interact with the substrate. As the nanostructure becomes thicker (≥100 nm), the transport takes place almost exclusively in the nanostructure. This leads to a saturation behavior of the distribution of the deposited energy, the charge, and the backscattered electrons. The simulations show two effects which may be important for the growth of the nanostructure. (1) The energy deposited in the substrate is available for the dissociation of precursor-gas molecules adsorbed on the surface substrate. (2) If we assume that a larger yield of secondary electrons enhances the dissociation of the precursor and improves the conditions for nanodeposit growth with high density and high metal content, then the simulations show that a larger deposit density leads to enhanced electron backscattering. This implies that random fluctuations in deposit density could be amplified through positive feedback.

The presented simulations therefore provide an overview of the effect of the primary electron beam on the deposit and on the substrate at different stages of the nanostructure growth. Furthermore, the distributions of the deposited energy serve as a starting point for further microscopic simulations (molecular dynamics) in that they provide a guideline for the initial temperature distribution in the substrate and the deposit under irradiation with an electron beam. Moreover, similar simulations can aid the understanding of the role that is played by backscattered and secondary electrons in changing the structural properties of nanostructured materials in several post-growth techniques, including the direct or oxygen-assisted electron-beam curing [5–6].

## References

[1] Koops H W P, Kretz J, Rudolph M, Weber M (1993). J Vac Sci Technol, B.

[2] Randolph S J, Fowlkes J D, Rack P D (2006). Crit Rev Solid State Mater Sci.

[3] Utke I, Hoffmann P, Melngailis J (2008). J Vac Sci Technol, B.

[4] Huth M, Porrati F, Schwalb C, Winhold M, Sachser R, Dukic M, Adams J, Fantner G (2012). Beilstein J Nanotechnol.

[5] Porrati F, Sachser R, Schwalb C H, Frangakis A S, Huth M (2011). J Appl Phys.

[6] Mehendale S, Mulders J J L, Trompenaars P H F (2013). Nanotechnology.

[7] Salvat F, Fernández-Varea J M, Sempau J (2011). PENELOPE-2011: A code System for Monte Carlo Simulation of Electron and Photon Transport.

[8] Joy D C (1995). Monte Carlo modelling for Electron Microscopy, Microanalysis.

[9] Lin Y, Joy D C (2005). Surf Interface Anal.

[10] Liu Z Q, Mitsuishi K, Furuya K (2005). Nanotechnology.

[11] Silvis-Cividjian N, Hagen C W, Leunissen L H, Kruit P (2002). Microelectron Eng.

[12] Smith D A, Fowlkes J D, Rack P D (2007). Nanotechnology.

[13] Smith D A, Fowlkes J D, Rack P D (2008). Small.

[14] Hoyle P C, Ogasawara M, Cleaver J R A, Ahmed H (1993). Appl Phys Lett.

[15] Hoyle P C, Cleaver J R A, Ahmed H (1996). J Vac Sci Technol, B.

[16] Huth M, Klingenberger D, Grimm C, Porrati F, Sachser R (2009). New J Phys.

[17] Beranová S, Wesdemiotis C (1994). J Am Soc Mass Spectrom.

[18] Cooks R G, Ast T, Kralj B, Kramer V, Žigon D (1990). J Am Soc Mass Spectrom.

[19] Michels G D, Flesch G D, Svec H J (1980). Inorg Chem.

[20] Cooper G, Green J C, Payne M P, Dobson B R, Hillier I H (1987). J Am Chem Soc.

[21] Hubbard J L, Lichtenberger D L (1982). J Am Chem Soc.

[22] Qi F, Yang S, Sheng L, Gao H, Zhang Y, Yu S (1997). J Chem Phys.

[23] Muthukumar K, Valentí R, Jeschke H O (2012). New J Phys.

[24] Salvat F, Jablonski A, Powell C J (2005). Comput Phys Commun.

[25] Bret T, Utke I, Bachmann A, Hoffmann P (2003). Appl Phys Lett.

[26] Plank H, Smith D A, Haber T, Rack P D, Hofer F (2012). ACS Nano.

[27] Randolph S J, Fowlkes J D, Rack P D (2005). J Appl Phys.

